# 

**Published:** 1877-06

**Authors:** 


☞ Read Notice of this Journal among advertisements.

Vol. XXXIV. Number 6.

**June, 1877.**

NEW SERIES, Vol. II. Number 6.

### THE CHICAGO MEDICAL Journal and Examiner

**EDITOR:**
WILLIAM H. BYFORD, A.M., M.D.

**ASSOCIATE EDITORS:**
JAMES H. ETHERIDGE, M.D. | JAS. NEVINS HYDE, A.M., M.D.
NORMAN BRIDGE, M.D. | FERD. C. HOTZ, M.D.

**ASSISTANT EDITOR:**
E. FLETCHER INGALS, M.D.

CHICAGO MEDICAL PRESS ASSOCIATION.

AUG. 2D 1875.

CHICAGO:
*PUBLISHED BY CHICAGO MEDICAL PRESS ASSOCIATION.*
188 Clark Street.

**Published Monthly. Terms—Four Dollars per annum, IN ADVANCE.**
**Single Copies, Thirty-Five Cents.**

**Subscriptions and Communications should be sent to the Assistant Editor, E. F. INGALS, M.D., 188 Clark Street.**

